# On-admission and dynamic trend of laboratory profiles as prognostic biomarkers in COVID-19 inpatients

**DOI:** 10.1038/s41598-023-34166-z

**Published:** 2023-04-28

**Authors:** Fatemeh Siavoshi, Seyed Amir Ahmad Safavi-Naini, Siavash Shirzadeh Barough, Mehdi Azizmohammad Looha, Hamidreza Hatamabadi, Davood Ommi, Reza Jalili Khoshnoud, Alireza Fatemi, Mohamad Amin Pourhoseingholi

**Affiliations:** 1 Basic and Molecular Epidemiology of Gastrointestinal Disorders Research Center, Research Institute for Gastroenterology and Liver Diseases, Shahid Beheshti University of Medical Sciences, Tehran, Iran; 2 Safety Promotion and Injury Prevention Research Center, Imam Hossein Hospital, Shahid Beheshti University of Medical Sciences, Tehran, Iran; 3 Department of Anesthesiology, School of Medicine, Shohada-e-Tajrish Hospital, Shahid Beheshti University of Medical Sciences, Tehran, Iran; 4 Department of Neurosurgery, School of Medicine, Shohada-e-Tajrish Hospital, Shahid Beheshti University of Medical Sciences, Tehran, Iran; 5 Men’s Health and Reproductive Health Research Center, Shahid Beheshti University of Medical Sciences, Tehran, Iran

**Keywords:** Biomarkers, Outcomes research

## Abstract

This large-scale study aimed to investigate the trend of laboratory tests of patients with COVID-19. Hospitalized confirmed and probable COVID-19 patients in three general hospitals were examined from March 20, 2020, to June 18, 2021. The confirmed and probable COVID-19 patients with known outcomes and valid laboratory results were included. The least absolute shrinkage and selection operator (LASSO) and Cox regression were used to select admittance prognostic features. Parallel Pairwise Comparison of mortality versus survival was used to examine the trend of markers. In the final cohort, 11,944 patients were enrolled, with an in-hospital mortality rate of 21.8%, mean age of 59.4 ± 18.0, and a male-to-female ratio of 1.3. Abnormal admittance level of white blood cells, neutrophils, lymphocytes, mean cellular volume, urea, creatinine, bilirubin, creatine kinase-myoglobin binding, lactate dehydrogenase (LDH), Troponin, c-reactive protein (CRP), potassium, and creatinine phosphokinase reduced the survival of COVID-19 inpatients. Moreover, the trend analysis showed lymphocytes, platelet, urea, CRP, alanine transaminase (ALT), and LDH have a dissimilar trend in non-survivors compared to survived patients. This study proposed a novel approach to find serial laboratory markers. Serial examination of platelet count, creatinine, CRP, LDH, and ALT can guide healthcare professionals in finding patients at risk of deterioration.

## Introduction

Since December 2019, COVID-19 has caused five disease surges and challenged healthcare systems worldwide. The World Health Organization (WHO) reported over 6,8 million fatalities because of COVID-19 on February 2023^1^, and the actual COVID-19 death toll may exceed twice the reported numbers^2^. Even after the development of vaccines, the COVID-19 virus continued to spread, and the mutations can challenge vaccine effectiveness and cause future peaks. The arrival of new sublineages, such as BA.5, suggests the prolonged impact of COVID-19 on healthcare systems, and solid evidence for investigating this disease is warranted^3^.

COVID-19 is a viral infection with several clinical presentations and complications. In addition to respiratory damage, inflammation can also damage parts of the human body, including kidneys, liver, nervous system, and reproductive systems^4^. The laboratory examination can be beneficial for assessing patient prognosis by detecting these multi-organ damages^5–7^. In addition, laboratory values can be disturbed in patients at higher risk of death and with comorbidities. Therefore, laboratory exams can help physicians distinguish poor prognosis patients on admission^7^.

Still, the dynamic changes in COVID-19 patients need investigation. A serial laboratory examination can guide healthcare professionals in the monitoring of patients. Limited studies used different methods to explain the dynamic change of COVID-19 inpatients^8–13^. Nonetheless, solid evidence is needed for choosing a practical set of laboratories to monitor patients. This large-scale study aimed to investigate the trend and admittance laboratory results of poor prognosis patients.

## Material and methods

### Ethics statement

The Shahid Beheshti University of Medical Science's ethical committee approved the study, waived obtaining informed consent, and obligated collaborators to anonymize patients’ identities (IR.SBMU.RIGLD.REC.1400.014). Data were collected retrospectively and anonymously, and data confidentiality was concerned. The declaration of Helsinki was addressed during the study.

### Study subjects

This retrospective cross-sectional study was conducted in three general hospitals in Tehran with a sum of 1120 beds (Taleghani Hospital, Emam Hossein Hospital, Shohada-E-Tajrish Hospital). All hospitalized confirmed and probable COVID-19 patients admitted from March 20, 2020, to June 18, 2021, were enrolled in the study. Patients who left the hospital for personal reasons, were under 18, were transferred to another hospital for further treatment, or had two negative COVID-19 reverse transcription polymerase chain reaction (RT-PCR) were excluded from the study. Age, gender, disease outcome (death, survived), ward of admission (emergency, COVID-19 ward, intensive care unit (ICU)), and laboratory values of COVID-19 patients were extracted from the hospital electronic information system (HEIS) and cleaned using pandas library of python programming language (Python Software Foundation. Python Language Reference, version 2.7. Available at http://www.python.org).

### Case definition

Hospitalized patients were retrieved from HEIS with international classification of diseases (ICD) codes of confirmed cases (U0.71) and probable cases (U07.2). Based on radiologic findings of COVID-19, clinical status, and the relevant specialist's opinion, suspected COVID-19 cases that did not underwent RT-PCR tests, due to the availability of diagnostic kits in Iran, were hospitalized as probable COVID-19 cases.

A “confirmed COVID-19 case” was defined as a person with positive RT-PCR for SARS-CoV-2, regardless of the presence of clinical signs and symptoms and close contact. A “probable COVID-19 case” was a suspected COVID-19 case: 1—with radiological findings that, according to radiologists, are strongly suggestive of the COVID-19 disease, such as one- or two-sided multilobular infiltration, especially infiltration of peripheral areas in a lung CT scan or chest radiograph and ground glass in lung CT scan (clinically confirmed), 2—with pneumonia who has an inappropriate clinical response despite proper treatment and the patient's clinical condition becomes more critical and worse or dies unusually and unexpectedly (clinically confirmed), 3—with inconclusive RT-PCR test result (The result of the person's RT-PCR test is unknown and is not reported as positive or negative.), or without any RT-PCR test due to any reason. Therefore, based on radiologic findings of COVID-19, clinical status, and the relevant specialist's opinion, suspected COVID-19 cases that did not undergo RT-PCR tests due to the lack or absence of diagnostic kits in Iran were hospitalized as probable COVID-19 cases.

A “suspected COVID-19 case” was considered as 1—a person with a history of dry cough or chills or sore throat with shortness of breath with or without fever that cannot be explained by another etiological factor, 2—a patient with fever or respiratory symptoms (of any severity) who is a healthcare staff or has a history of close contact with a probable/definite COVID-19 case within 14 days before the onset of the disease symptoms.

Radiological findings highly suggestive of COVID-19 pneumonia were used to find probable cases in some time periods. Iran's national guideline for diagnosing and treating COVID-19 and the Iranian Society of Radiology COVID-19 Consultants (ISRCC) defined radiologic findings of COVID-19. Existence of unilateral/bilateral, unifocal/multifocal ground glass opacity (GGO), consolidation or nodules more likely with peripheral distribution or less likely with periburonchovascular distribution, diffuse/bilateral infiltrates of the lungs consistent with ARDS, patchy consolidation with surrounding ground glass halo (late finding), patchy consolidation with and without air-bronchogram, crazy-paving appearance (late finding), peripheral stripe like opacities (late finding) identified as the typical CT features of COVID-19 on thorax CT.

### Study setting

This study had been conducted in three tertiary general hospitals located at the North, East, and West of Tehran with, a total of 1200 hospital-beds. During disease peaks, multiple wards were allocated for care of COVID-19 inpatients. At the time of the study, the dominant COVID-19 variants in Iran were Alpha and Beta, which are fairly similar patients' characteristics, management, or outcomes^14^. Therefore, patients were not classified based on the COVID-19 variants. In addition, COVID-19 vaccination started in April 2021 for the general population, and about 1.08% of total population was immunized against COVID-19^15^. Given these points, there were a few vaccinated patients referred to the hospital who were not included in the study.

### Admittance and serial lab exam

Laboratory values included for analysis were as follows: Complete blood count (CBC), cardiac, liver, pancreatic, thyroid, renal, electrolytes, blood glucose, and inflammatory profiles, coagulation, lipid, and iron profile, venous blood gas (VBG), vitamin D3, immunoglobin M (IgM), and immunoglobin G (IgG) levels.

The valid laboratory results for the first six days of admission were sorted for 12 lab values, including white blood cell (WBC), lymphocyte count, neutrophil count, platelet (PLT), hemoglobin (Hb), mean corpuscular volume (MCV), BUN (calculated by dividing urea by 2.14), creatinine (Cr), aspartate transaminase (AST), alanine transaminase (ALT), lactate dehydrogenase (LDH), C-reactive protein (CRP). Daily serial laboratory values during admission were investigated in patients with at least four valid laboratory values during the six first days of admission.

### Statistical analysis

Statistical data analyses and visualization were performed using the R program 4.1.1 (Packages: dplyr, caret, tidyverse, FactoMineR, outliers, ggpubr, ggplot, ggrepel). Continuous variables were presented as mean ± standard deviation, while categorical variables were expressed as frequencies and percentages. The Mann–Whitney U and Fisher’s exact tests were performed to compare the differences between groups for continuous and categorical variables, respectively. The least absolute shrinkage and selection operator (LASSO) regression model was used to select prognostic features. Cox regression was used to find patients’ mortality hazard ratio for confirmed features by LASSO regression. For variables with significant differences between survival and non-survival groups but with hazard ratio (HR) close to one, we performed logarithmic transformation to improve their interpretability. Kaplan–Meier survival analysis and the Log-rank test of the mortality risk factors were performed to assess the survival probability during 60-day hospitalization.

The data points higher than 3 or lower than -3 of the sample Z-score were considered outliers. The normalization of laboratory values was investigated by the Q–Q plot, and the Wilcoxon signed-rank test was used to investigate the difference in means of serial laboratory values when the data were non-parametric. For parametric data, paired t-test was used. Joint point program 4.9.0 was used to perform the Parallel Pairwise Comparison of mortality versus survival for serial laboratory values^16,17^. The Parallel Pairwise Comparison investigates the significance level for the test of coincidence or parallelism. The average daily percent change, confidence interval, and *p*-value for the trend of laboratory values of 6 days of admission were obtained.

## Results

Of the total 14,045 COVID-19 patients, 11,944 were included in this study, of which 7417 subjects were hospitalized as confirmed COVID-19 cases with positive RT-PCR tests and 4527 as probable COVID-19 cases with no RT-PCR tests. There were 9342 survivors and 2602 non-survivors, with an in-hospital mortality rate of 21.8% in the entire cohort. Moreover, the mortality rate was higher for those needing ICU care (63.1%). The mean age of patients was 59.4 ± 18.0, with a range of 18–104, and 56.8% of patients were male.

### Baseline on-admission laboratory parameters

The baseline laboratory parameters of the COVID-19 patients on admission are summarized in Table 1. The two groups significantly differed in laboratory parameters indicating inflammation, tissue necrosis, organ dysfunction, and coagulation disorders (*p*-value < 0.001). Abnormal electrolytes, VBG, and vitamin D3 levels were significantly higher in the non-survivors group. Also, non-survivors had more elevated cholesterol and fasting blood sugar (FBS) levels. Still, the two groups had no significant differences in other lipid profiles indicating dyslipidemia and the HbA1C level. Moreover, IgM and IgG levels were not associated with disease severity. As shown in Table 2, nineteen variables were finally identified as potential risk factors for mortality.

**Table 1:** Basic characteristics of survivors COVID-19 (N = 9342) and non-survivors (N = 2602).

	Total Mean ± SD n/N(%)	Survivors Mean ± SD n (%)	Non-survivors Mean ± SD n (%)	*p* value
Gender				0.001
Male	6779/11,944 (56.8%)	5229/9342(56.0%)	1550/2602 (59.6%)	
Female	5165/11,944 (43.2%)	4113/9342 (44.0%)	1052/2602 (40.4%)	
Age (years)	59.4 ± 18.0	56.3 ± 17.4	70.4 ± 15.7	< 0.001
18–49	3687 (30.9%)	3402 (36.4%)	285 (11.0%)	< 0.001
50–62	2804 (23.5%)	2361 (25.3%)	443 (17.0%)	< 0.001
63–75	2873 (24.1%)	2138 (22.9%)	735 (28.2%)	< 0.001
76–104	2580 (21.6%)	1441 (15.4%)	1139 (43.8%)	< 0.001
Hospitalization (days)	6.4 ± 7.5	5.9 ± 6.8	8.1 ± 9.4	< 0.001
Hospitalization ward (%)				
Emergency	2292(19.2%)	2133 (22.8%)	159 (6.1%)	< 0.001
ward	7772(65.1%)	6516 (69.7%)	1256 (48.3%)	
ICU	1880 (15.7%)	693 (7.4%)	1187 (45.6%)	
Hematologic parameters				
Leukocytes (normal range 3.5–9.5 × 10^9^/L)	8.4 ± 6.0	7.8 ± 4.8	10.9 ± 8.8	** < 0.001**
Leukocyte count < 3.5 × 10^9^/L	735/10,537 (7.0%)	599 (7.3%)	136 (5.9%)	** < 0.001**
Leukocyte count > 9.5 × 10^9^/L	3113/10,537(29.5%)	2008 (24.5%)	1105 (47.7%)	** < 0.001**
Lymphocyte percentage (Normal range 20–50)	19.1 ± 11.3	20.7 ± 11.2	13.5 ± 9.6	** < 0.001**
Lymphocyte (normal range 1.1–3.2 × 10^9^/L)	1257.6 ± 1965.3	1262.8 ± 1436.0	1238.9 ± 3214.2	0.583
Lymphocyte count < 1.2 × 10^9^/L	6992/11,942(58.5%)	5229 (56.0%)	1763 (67.7%)	** < 0.001**
Neutrophils percentage (Normal range 40–75)	75.9 ± 12.9	74.2 ± 12.8	82.3 ± 11.0	** < 0.001**
Neutrophils (normal range1.5–6.3 × 10^9^/L)	5734.4 ± 4834.3	5143.1 ± 3962.7	7875.6 ± 6730.7	** < 0.001**
Neutrophils count < 1.5 × 10^9^/L	1739/11,933(14.6%)	1376 (11.5%)	365 (3.9%)	** < 0.001**
Neutrophils count > 6.3 × 10^9^/L	4206/11,942(35.2%)	2788 (29.8%)	1418 (54.5%)	** < 0.001**
Neutrophil-to-lymphocyte ratio	6.4 ± 5.8	5.5 ± 4.9	9.5 ± 7.4	** < 0.001**
Neutrophil-to-lymphocyte ratio > 7.726	2993/10,377(28.8%)	1816 (22.4%)	1177 (52.1%)	** < 0.001**
Platelets (normal range150-450 × 10^9^/L)	209.4 ± 91.7	210.9 ± 87.1	204.0 ± 106.4	**0.002**
Platelets < 150 × 10^9^/L	2528/10,344(24.4%)	1815 (22.5%)	713 (31.4%)	** < 0.001**
Platelets > 450 × 10^9^/L	187/10,344(1.8%)	133 (1.6%)	54 (2.4%)	** < 0.001**
HB (normal range male: 13.5–17.5 g/dL, female: 12.5–15.5 g/dL)	12.3 ± 2.4	12.5 ± 2.2	11.7 ± 2.6	** < 0.001**
HB < 12.55 g/dL (female) & HB < 13.55 g/dL (male)	5042/10,495(48.0%)	3635 (44.4%)	1407 (61.1%)	** < 0.001**
MCV (normal range 80–100 fL)	83.8 ± 7.5	85.2 ± 7.8	83.4 ± 7.4	** < 0.001**
MCV < 80 fL	2452/10,509(23.3%)	2003 (24.4%)	449 (19.5%)	** < 0.001**
MCV > 100 fL	108/10,509 (1.0%)	61 (0.7%)	47 (2.0%)	** < 0.001**
Kidney function				
BUN (normal range 6–23 mg/dL)	25.7 ± 22.2	21.6 ± 15.9	39.8 ± 32.4	** < 0.001**
BUN > 23 mg/dL	1736/4915 (35.3%)	1018 (26.8%)	718 (64.5%)	** < 0.001**
Cr (normal range 0.5–1.2 mg/dL)	1.5 ± 1.4	1.4 ± 1.2	2.0 ± 1.9	** < 0.001**
Cr > 1.2 mg/dL	4534/9721 (46.6)	3028 (40.6%)	1506 (66.3%)	** < 0.001**
Electrolytes parameters				
Na (normal range 135–145 mmol/L)	138.3 ± 13.5	138.4 ± 14.9	138.2 ± 6.9	0.632
Na < 135 mmol/L	1945/10,742 (18.1%)	1346 (16.3%)	599 (23.9%)	** < 0.001**
Na > 145 mmol/L	495/10,742 (4.6%)	273 (3.3%)	222 (8.9%)	** < 0.001**
K(normal range 3.5–5.1 mmol/L)	4.2 ± 1.9	4.2 ± 2.0	4.3 ± 1.3	** < 0.001**
K < 3.5 mmol/L	796/10,789 (7.4%)	569 (6.9%)	227 (9.0%)	** < 0.001**
K > 5.1 mmol/L	583/10,789 (5.4%)	298 (3.6%)	285 (11.3%)	** < 0.001**
Ca (normal range 8.5–10.5 mg/dL)	8.9 ± 11.7	9.0 ± 13.8	8.5 ± 1.1	0.085
Ca < 8.5 mg/dL	3246/8082 (40.2%)	2109 (35.9%)	1137 (51.4%)	** < 0.001**
Ca > 10.1 mg/dl	621/8082 (7.7%)	469 (8.0%)	152 (6.9%)	** < 0.001**
Mg (normal range 1.3–2.1 mEq/L)	2.0 ± 0.4	2.0 ± 0.3	2.0 ± 0.4	** < 0.001**
Mg < 1.6 mg/dl	622/8141 (7.6%)	431 (7.3%)	191 (8.7%)	** < 0.001**
Mg > 2.4 mg/dl	716/8141 (8.8%)	422 (7.1%)	294 (13.3%)	** < 0.001**
P (normal range 3.0–4.5 mg/dL)	3.6 ± 1.3	3.4 ± 1.0	4.1 ± 1.7	** < 0.001**
P < 2.5 mg/dl	778/6431 (12.1%)	588 (12.7%)	190 (10.6%)	** < 0.001**
P > 4.5 mg/dl	933/6431 (14.5%)	431 (9.3%)	502 (28.1%)	** < 0.001**
Liver function				
AST (normal range 0-37 IU/L(	67.3 ± 218.9	53.9 ± 154.1	106.5 ± 341.6	** < 0.001**
AST > 37 IU/L	2990/6245 (47.9%)	2002 (43.0%)	988 (62.1%)	** < 0.001**
ALT(normal range 4–40 IU/L(	55.0 ± 174.5	46.6 ± 114.4	79.6 ± 283.7	** < 0.001**
ALT > 40 IU/L	2039/6240 (32.7%)	1442 (31.0%)	597 (37.5%)	** < 0.001**
ALP (normal range 30–150 IU/L)	226.8 ± 194.1	212.5 ± 156.7	267.7 ± 270.0	** < 0.001**
ALP > 150 IU/L	6120/8780 (69.7%)	4432 (68.2%)	1688 (74.1%)	** < 0.001**
Total Bilirubin (normal range 0.3–1.2 mg/dL)	1.0 ± 1.8	0.9 ± 1.0	1.4 ± 3.1	** < 0.001**
Total Bilirubin > 1.2	1543/7600 (20.3%)	957 (17.1%)	586 (29.1%)	** < 0.001**
Direct Bilirubin (normal range 0.1–0.3 mg/dL)	0.4 ± 1.1	0.4 ± 0.6	0.7 ± 1.9	** < 0.001**
Direct Bilirubin > 0.3 mg/dL	3721/7577 (49.1%)	2427 (43.6%)	1294 (64.5%)	** < 0.001**
Albumin (normal range 3.5–5.5 g/dL)	3.8 ± 0.8	3.9 ± 0.7	3.5 ± 0.9	** < 0.001**
Albumin < 3.5 g/dL	1881/6572 (28.6%)	939 (20.0%)	942 (50.0%)	** < 0.001**
Pancreatic function				
Amylase (normal range 60–120 Somogyi U/dL)	75.5 ± 109.3	70.3 ± 94.6	89.0 ± 139.7	** < 0.001**
Amylase > 120 Somogyi U/dL	246/2472 (9.9%)	137 (7.7%)	109 (15.9%)	** < 0.001**
Lipase (normal range 0–160 IU/L(	38.5 ± 71.4	36.2 ± 59.4	44.5 ± 95.8	**0.011**
Lipase > 160 IU/L	39/2352 (1.7%)	27 (1.6%)	12 (1.9%)	0.718
Lipid profile				
TGL(normal range < 200 mg/dL)	144.3 ± 94.8	142.9 ± 93.8	148.7 ± 97.8	0.312
TGL > 200 mg/dL	243/1465 (16.6%)	180 (16.3%)	6 (17.6%)	0.684
Cholesterol(normal range < 200 mg/dL)	136.2 ± 43.0	139.1 ± 43.3	127.3 ± 40.7	** < 0.001**
Cholesterol > 200 mg/dL	77/1441 (5.3%)	49 (4.5%)	28 (7.9%)	**0.032**
HDL(normal range > 55 mg/dL)	34.3 ± 11.7	34.7 ± 11.2	32.9 ± 13.1	0.200
HDL < 55 mg/dL	1277/1325 (96.4%)	974 (96.2%)	303 (97.1%)	0.492
LDL(normal range < 130 mg/dL)	78.7 ± 31.4	81.0 ± 31.5	71.3 ± 30.0	** < 0.001**
LDL > 130 mg/dL	57/1319 (4.3%)	37 (3.6%)	20 (6.4%)	0.097
Blood glucose				
FBS (normal range 74–110 mg/dL)	161.1 ± 84.6	156.8 ± 82.3	171.7 ± 89.2	** < 0.001**
FBS > 110 mg/dL	2174/3148 (69.1%)	1501 (66.9%)	673 (74.5%)	** < 0.001**
HbA1C(normal range 5.7–6.4 )	8.1 ± 2.3	8.1 ± 2.3	8.1 ± 2.2	0.869
HbA1C > 6.4	1599/2108 (75.9%)	1188 (75.7%)	411 (76.4%)	0.771
Inflammatory markers				
LDH (normal range < 250 IU/L)	686.0 ± 598.5	607.6 ± 452.3	918.4 ± 861.6	** < 0.001**
LDH > 250 IU/L	4422/4538 (97.4%)	3296 (97.1%)	1126 (98.4%)	**0.017**
CRP (normal range 0-10 mg/L)	43.0 ± 47.6	38.7 ± 44.9	58.3 ± 53.3	** < 0.001**
CRP > 10 mg/L	6284/8479 (74.1%)	4702 (71.1%)	1582 (84.8%)	** < 0.001**
ESR (normal range ≤ 15 mm/hr)	37.3 ± 25.6	35.8 ± 24.8	41.7 ± 27.4	** < 0.001**
ESR > 15 mm/hr	6880/8854 (77.7%)	5081 (76.3%)	1799 (82.0%)	** < 0.001**
Lactate (normal range 4.5–19.8 mg/dL)	24.1 ± 18.0	21.3 ± 10.9	29.1 ± 25.5	** < 0.001**
Lactate > 19.8 mg/dL	1541/3016 (51.1%)	898 (46.7%)	643 (58.8%)	** < 0.001**
IL6	78.2 ± 128.6	64.9 ± 112.6	103.1 ± 151.5	**0.003**
CPK (normal range ≤ 200 IU/L)	315.1 ± 1050.1	264.3 ± 796.1	471.2 ± 1584.8	** < 0.001**
CPK > 200 IU/L	2905/9484 (30.6%)	1933 (27.0%)	972 (41.7%)	** < 0.001**
Procalcitonin (normal range ≤ 0.1 ng/mL)	2.6 ± 7.6	1.7 ± 6.3	4.0 ± 9.2	** < 0.001**
Procalcitonin > 0.1 ng/mL	1122/1334 (84.1%)	674 (80.2%)	448 (90.7%)	** < 0.001**
Cardiac function				
Troponin (normal range < 0.04 ng/mL)	0.6 ± 6.8	0.3 ± 3.7	1.2 ± 10.8	** < 0.001**
Troponin > 0.04 ng/mL	1357/4838 (28.0%)	604 (18.2%)	753 (49.9%)	** < 0.001**
CK-MB (normal range 3–5%)	39.7 ± 119.9	32.9 ± 64.1	59.2 ± 207.8	** < 0.001**
CK-MB > 5%	7097/7153 (99.2%)	5259 (99.3%)	1838 (99.1%)	0.446
proBNP(normal range < 300 pg/mL)	3826.9 ± 6939.0	2508.7 ± 5185.9	6618.4 ± 9046.1	** < 0.001**
proBNP > 300 pg/mL	894/1350 (66.2%)	512 (55.8%)	382 (88.2%)	** < 0.001**
Coagulation function				
aPTT (normal range 30–40 s)	32.3 ± 10.1	31.9 ± 10.1	33.3 ± 10.0	** < 0.001**
aPTT > 40 s	937/8780 (10.7%)	608 (9.4%)	329 (14.3%)	** < 0.001**
INR (normal range < 1.1)	1.2 ± 0.4	1.1 ± 0.3	1.3 ± 0.5	** < 0.001**
INR > 1.1	3261/8883 (36.7%)	1976 (30.2%)	1285 (55.1%)	** < 0.001**
PT (normal range 11–12.5 s)	13.3 ± 2.8	13.0 ± 2.3	14.1 ± 3.7	** < 0.001**
PT > 12.5 s	2358/8844 (26.7%)	1364 (20.9%)	994 (43.1%)	** < 0.001**
D-Dimer (normal range < 250 ng/mL)	1143.7 ± 1501.4	952.2 ± 1248.4	1700.3 ± 1965.3	** < 0.001**
D-Dimer > 250	3352/4215 (79.5%)	2419 (77.1%)	933 (86.5%)	** < 0.001**
VBG				
PH (normal range 7.31–7.41)	8.1 ± 71.6	8.3 ± 82.0	7.3 ± 0.2	0.557
PH < 7.31	1799/10,503 (17.1%)	1030 (12.9%)	769 (30.6%)	** < 0.001**
PCO2(normal range 40–50 mm Hg)	45.2 ± 12.2	45.4 ± 10.2	44.4 ± 17.2	**0.001**
PCO2 < 40 mm Hg	3154/10,476 (30.1%)	2141 (26.9%)	1013 (40.3%)	** < 0.001**
HCO3(normal range 22–28 mEq/L)	25.6 ± 5.6	26.2 ± 5.2	23.4 ± 6.2	** < 0.001**
HCO3 < 22 mEq/L	2166/10,466 (20.7%)	1229 (15.4%)	937 (37.3%)	** < 0.001**
Iron profile				
Serum Iron(normal range 60-170mcg/dL)	64.6 ± 64.3	64.6 ± 64.9	64.8 ± 62.6	0.962
Serum Iron < 60 mcg/dL	1004/1605(62.5%)	715 (61.9%)	289 (64.4%)	0.359
Ferritin (normal range 10–300 ng/mL)	424.8 ± 339.0	395.2 ± 320.8	523.1 ± 377.4	** < 0.001**
Ferritin > 300 ng/mL	2597/4687(55.4%)	1870 (51.9%)	727 (67.1%)	** < 0.001**
TIBC (normal range 240–450 mcg/dL)	262.7 ± 94.5	271.2 ± 89.4	241.3 ± 103.4	** < 0.001**
TIBC > 450 mcg/dL	30/1430 (2.1%)	18 (1.8%)	12 (2.9%)	0.157
Thyroid function				
TSH (normal range 0.32–5.2 mIU/L)	2.1 ± 4.9	2.0 ± 5.1	2.1 ± 4.5	.791
TSH < 0.32 mIU/L	287/1623(17.7%)	204 (17.1%)	83 (19.3%)	0.167
TSH > 5.2 mIU/L	115/1623 (7.1)	78 (6.5%)	37 (8.6%)	0.167
T4	8.2 ± 2.7	8.5 ± 2.6	7.2 ± 2.7	** < 0.001**
T3	0.9 ± 0.4	1.0 ± 0.4	0.8 ± 0.3	** < 0.001**
Vit D3 (normal range 25–80 ng/mL)	30.4 ± 20.9	29.9 ± 20.5	31.7 ± 21.9	**0.031**
Vit D3 < 25 ng/mL	1411/2937(48.0)	986 (46.7%)	425 (51.6%)	**0.017**
IgM (normal range 55–375 mg/dL)	85.0 ± 57.7	94.8 ± 61.3	55.2 ± 30.0	**0.013**
IgM < 55 mg/dL	21/69(30.4)	13 (25.0%)	8 (47.1%)	0.128
IgG (normal range 565–1765 mg/dL)	1231.1 ± 564.6	1274.8 ± 577.2	1110.0 ± 517.9	0.172
IgG < 565 mg/dL	6/113(5.3%)	3 (3.6%)	3 (10.0%)	0.189

Data are presented as means ± SDs or N (%). *ICU* Intensive care unit.

Significant values are in bold.

**Table 2:** Prognostic factors associated with mortality of COVID-19 cohort.

Item	HR (95%CI)	*p* value
Age		
18–49	1.0	–
50–62	1.6 (1.4, 1.9)	< 0.001
63–75	2.3 (2.0, 2.7)	< 0.001
76–104	4.3 (3.8, 4.9)	< 0.001
Male gender	1.1 (1.0, 1.2)	0.012
WBC < 3500	1.2 (1.0, 1.4)	0.033
WBC > 9500	1.8 (1.7, 2.0)	< 0.001
Neutrophils count > 6300	0.6 (0.5, 0.6)	< 0.001
Lymphocyte count < 1200	1.1 (1.0, 1.2)	0.009
MCV < 80	0.8 (0.8, 0.9)	0.001
CRP > 10	1.4 (1.2, 1.6)	< 0.001
PCO2 > 50	1.0 (0.9, 1.1)	0.775
Cr > 1.2	2.1 (2.0, 2.3)	< 0.001
BUN > 23	2.9 (2.6, 3.3)	< 0.001
Potassium > 5.1	2.1 (1.9, 2.4)	< 0.001
PT > 12.5	1.1 (1.0, 1.1)	< 0.001
Log ALP*	1.4 (1.3, 1.5)	< 0.001
Total bilirubin > 1.2	1.6 (1.4, 1.7)	< 0.001
Direct bilirubin > 0.3	1.7 (1.5, 1.9)	< 0.001
CPK > 200	1.4 (1.3, 1.6)	< 0.001
Log CK-MB*	1.4 (1.3, 1.4)	< 0.001
LDH > 250	1.2 (0.8, 1.9)	0.404
Troponin > 0.04	2.5 (2.3, 2.8)	< 0.001
D-DIMER > 250	1.4 (1.2, 1.7)	< 0.001

*Footnote* The Cox regression was used to analyze the impact of the variable on time to death of patients with COVID-19. *MCV* Mean corpuscular volume, *CRP* C-reactive protein, *Cr* Creatinine, *BUN* Blood urea nitrogen, *PT* Prothrombin time, *ALP* Alkaline phosphatase, *CPK* Creatine kinase, *LDH* Lactate dehydrogenase.

*The CK-MB and ALP are presented in logarithmic scale since the non-logarithmic hazard ratio is close to 1, and logarithmic transformation was used to facilitate interpretation.

### Survival analysis

**Figure 1:**
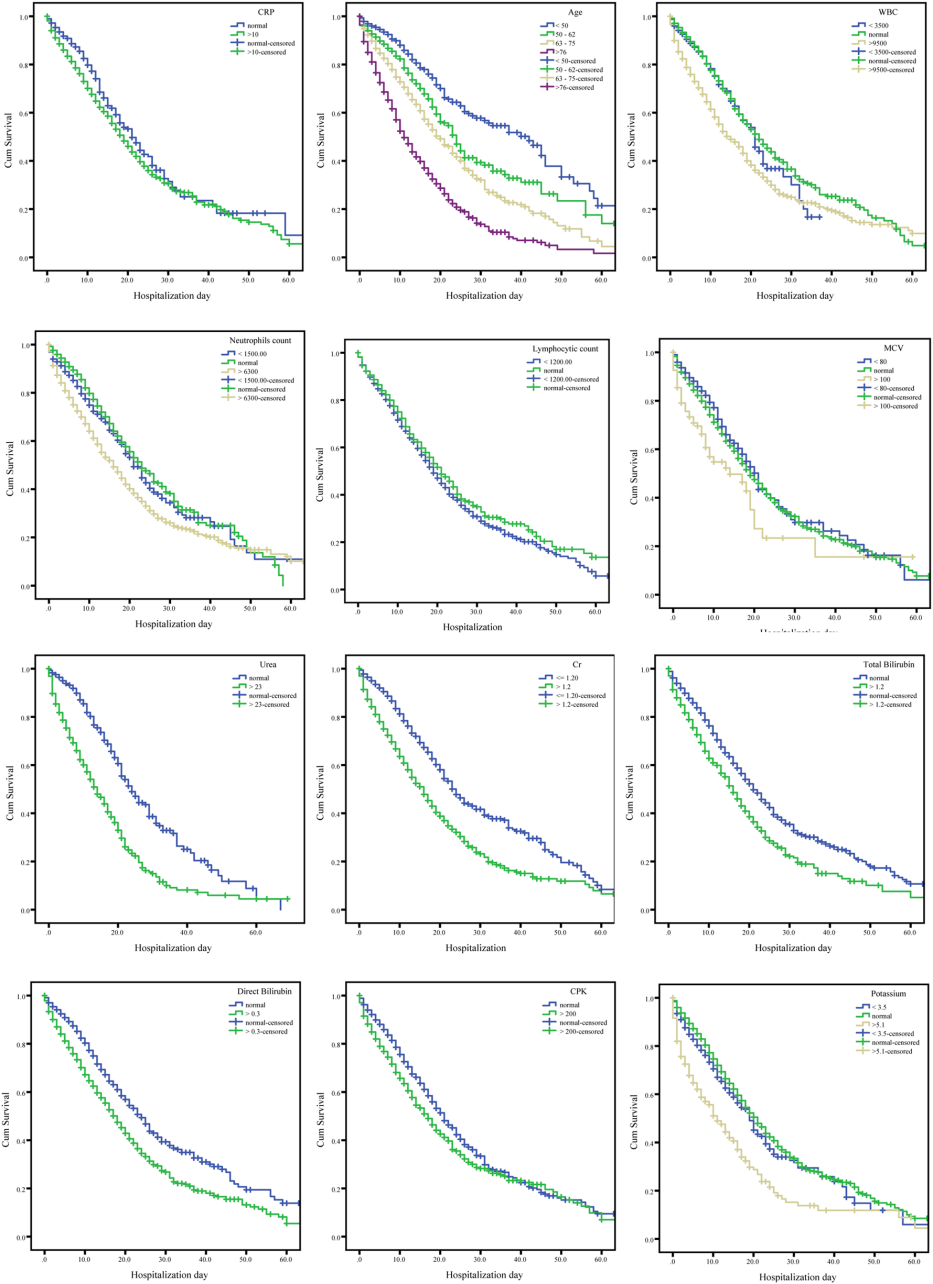
Survival curves of prognostic factors and admittance lab results of COVID-19 in hospitalized patients. (*Footnote* Abbreviations: *CRP* C-reactive protein; *WBC* White blood cell; *MCV* Mean corpuscular volume; *Cr* Creatinine; *CPK* Creatine phosphokinase).

Older age, male sex, and abnormal laboratory parameters at admission, including increased WBC count, decreased neutrophils and lymphocytic count, increased MCV, abnormal levels of BUN, Cr, total and direct bilirubin, Log CK-MB, LDH, Troponin, CRP, potassium (K), and creatinine phosphokinase (CPK) significantly associated with the reduced survival of COVID-19 patients (*p*-value < 0.05). However, according to Log-rank analysis, increased LDH did not significantly affect 60-day mortality in COVID-19 patients (*p*-value: 0.390). The survival curves of these laboratory parameters are shown in Fig. 1.

### Serial laboratory values

**Figure 2:**
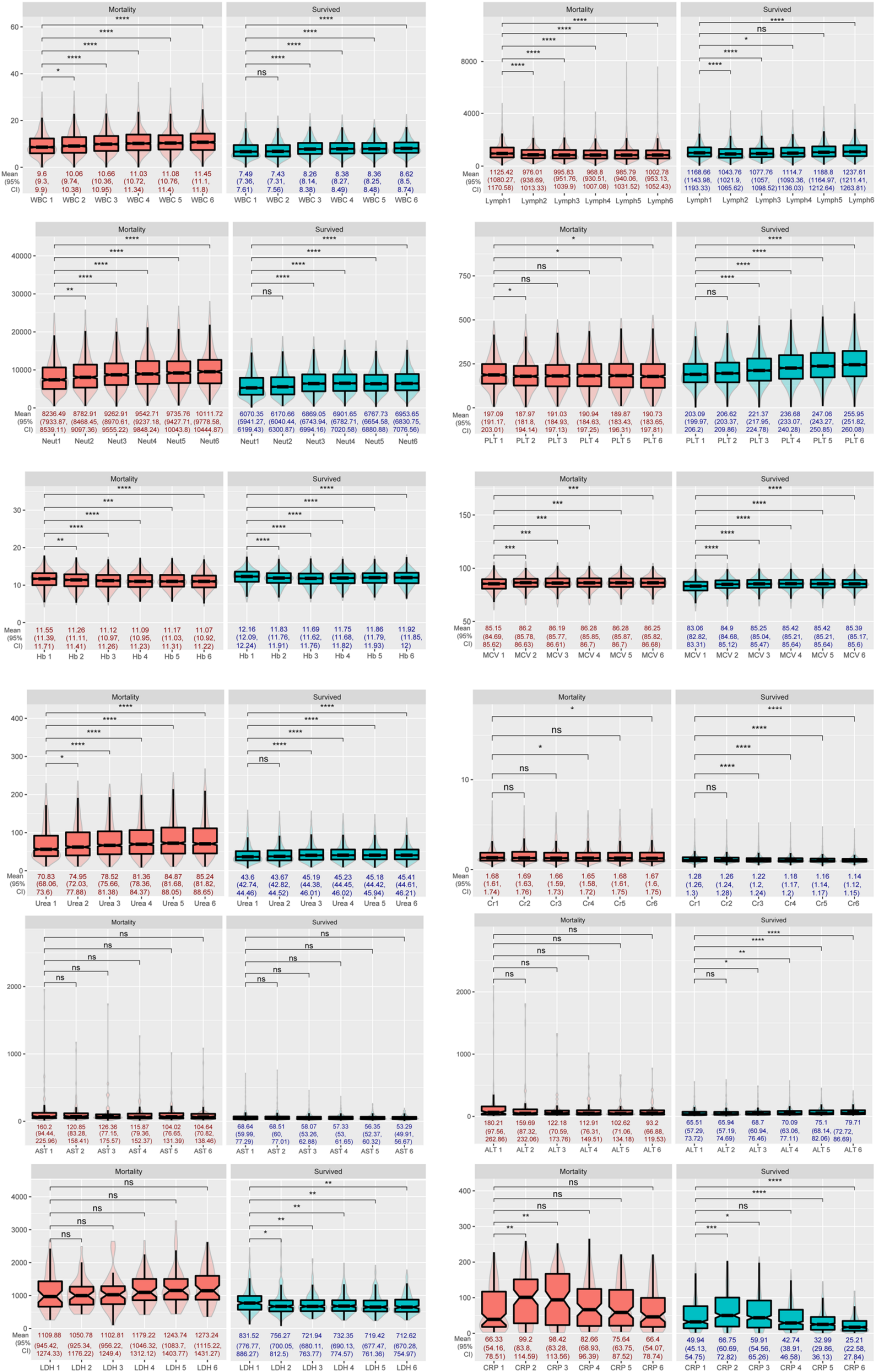
Serial laboratory values among COVID-19 inpatients (*Footnote* Wilcoxon signed-rank test used to evaluate *p*-value for all presented lab values. After outlier removal and selecting patients with a minimum of 4 valid data point number of patients are as follow (Mortality vs. Survived): WBC: 1284 vs. 4018, lymphocyte count: 927 vs. 2984, Neutrophil count: 932 vs. 2984, PLT: 950 vs. 3005, Hb: 964 vs. 3042, MCV: 972 vs. 3062, Urea: 1101 vs. 3571, Cr: 1097 vs. 3559, AST: 100 vs. 428, ALT: 99 vs. 424, LDH: 62 vs. 226, CRP: 103 vs. 417. Abbreviations: *WBC* White blood cell; *Lymph* Lymphocyte count; *Neut* Neutrophil count; *PLT* Platelet; *Hb* Hemoglobin; *MCV* Mean corpuscular volume; *Cr* Creatinine; *AST* Aspartate transaminase; *ALT* Alanine transaminase; *LDH* Lactate dehydrogenase; *CRP* C-reactive protein).

**Figure 3:**
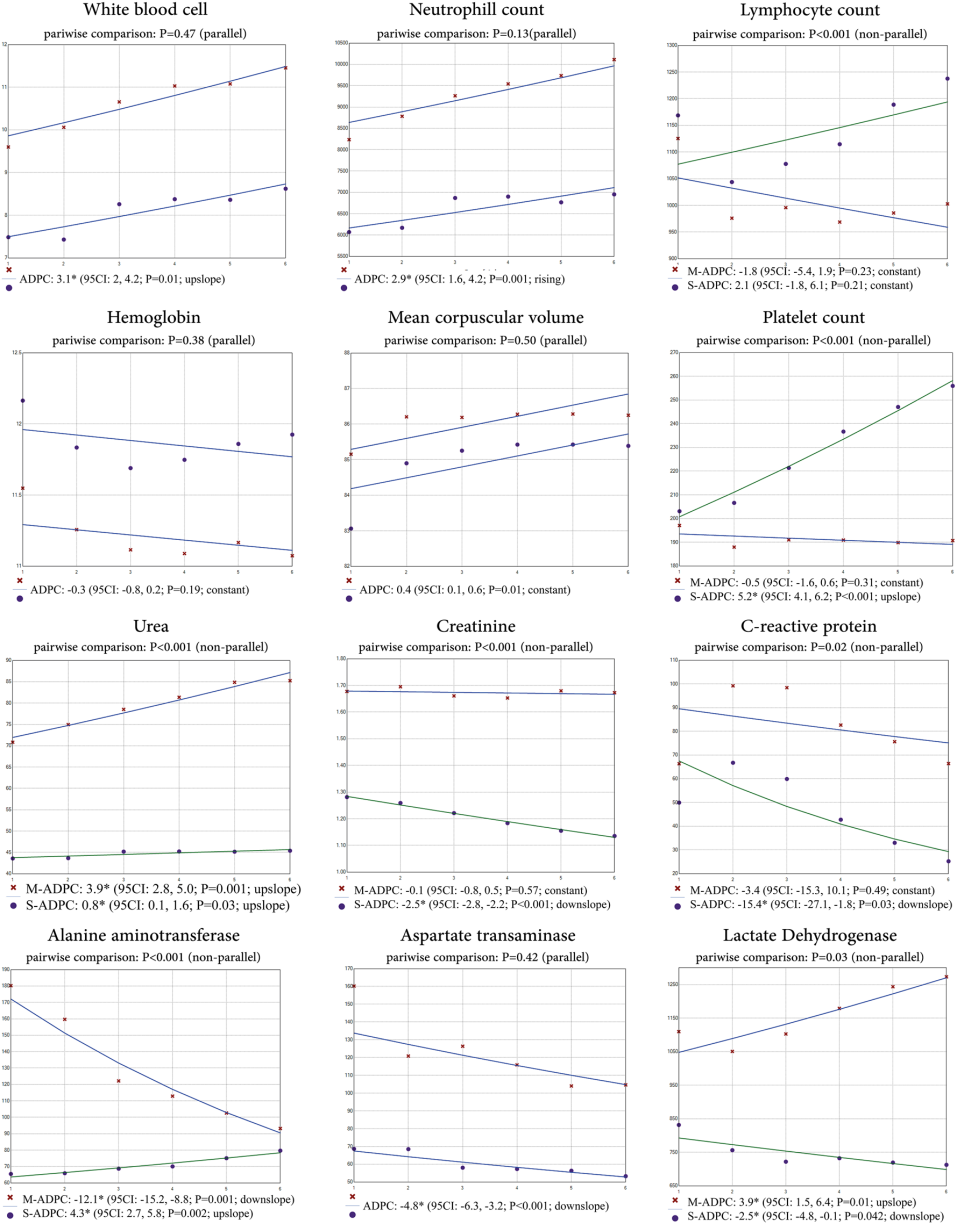
The joinpoint pairwise comparison (parallel) analysis of laboratory trends in survived COVID-19 inpatients versus patients who did not survive. (*Footnote* Red cross: Mortality; Blue dot: Survived. Abbreviations: *ADPC* Average daily percent change, 95CI 95% confidence interval, *M-ADPC* ADPC for mortality cohort, *S-ADPC* ADPC for the survived cohort).

The lab results during the first six days of admission are presented in Fig. 2. The pairwise comparison of the laboratory values’ trends between patients who survived and did not survive is depicted in Fig. 3. The trend of WBC (*p* = 0.47), neutrophil count (*p* = 0.13), Hb (*p* = 0.38), MCV (*p* = 0.5), and AST (*p* = 0.42) were parallel. However, lymphocyte count (*p* < 0.001), PLT count (*p* < 0.001), urea (*p* < 0.001), Cr (*p* < 0.001), CRP (*p* = 0.02), ALT (*p* < 0.001), and LDH (*p* = 0.03) were not parallel. The average daily percent change and *p*-value of trends are presented in Fig. 3.

## Discussion

**Figure 4:**
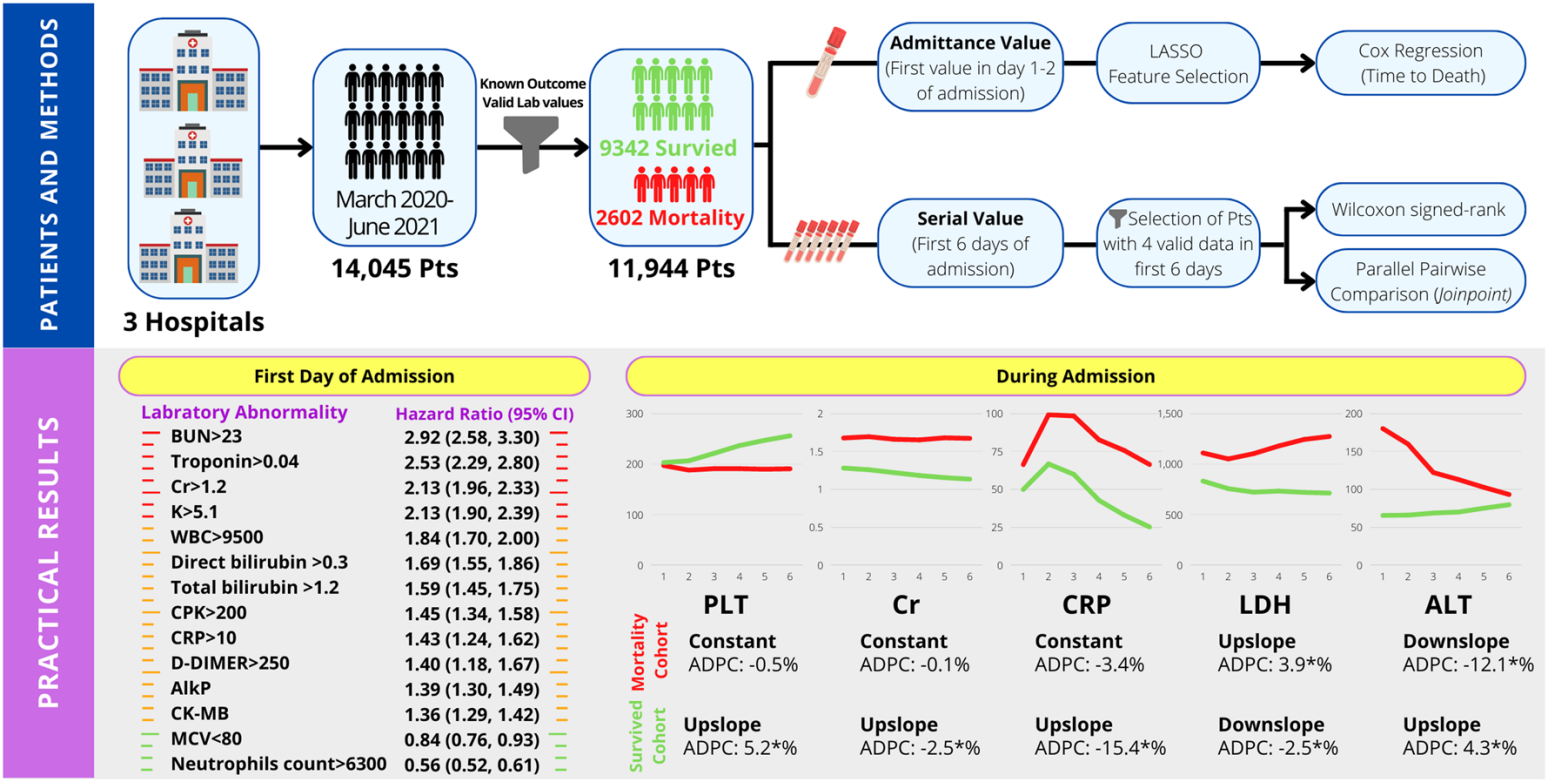
Summary of study method and population.

The laboratory parameters can be valuable for patient allocation and treatment protocol. This study investigated the laboratory profile of 11,944 patients with COVID-19 since the onset of the pandemic using the parallel pairwise comparison of Joinpoint—a novel approach for analyzing longitudinal laboratory results. We found that serial examination of PLT, Cr, CRP, LDH, and ALT can distinguish patients with poor prognoses, and these parameters can help monitoring the patient’s condition. There were significant differences in the admittance level of most laboratory parameters between the non-survived and survived cohorts. However, we just found abnormal WBC, MCV, BUN, Cr, total bilirubin, direct bilirubin, CRP, K, CPK, increased neutrophils, and decreased lymphocytic count as indicators of COVID-19 mortality. Figure 4 demonstrates a summary of the study method and practical findings.

Monitoring and allocating inpatients during peaks of COVID-19 can be challenging. Studies tried to propose laboratory profile for monitoring a hospitalized patient using longitudinal weekly values^9^, comparing early and late results^10^, historical regression tree^11^, Wilcoxon sum rank test of daily averages trend^8^, Mann–Whitney test of daily results between mortality and survived^12^, and dynamic time wrapping analysis^13^. This study used the parallel pairwise comparison to investigate the trend of lab results among survivors and non-survivors. If a lab trend is parallel between survived and mortality groups, it may fail to distinguish prognosis. Unparallel trend, especially with an inverse direction, may suggest that the trend can help finding patients at risk. In addition, the average daily percent change and the trend were explored in the case of non-parallel trends.

The WBC, neutrophil count, Hb, MCV, and AST had a parallel trend in the non-survived and survived cohorts. Although urea and lymphocyte count had a non-parallel trend, the trend was the same for the non-survived and survived cohorts (urea: both trends are upslope, lymphocyte count: both trends are constant). Finally, for PLT, Cr, ALT, CRP, and LDH, the trend of the non-survived and survived cohorts was non-parallel and distinguishable. Particularly, a rise in PLT (Average daily percent change (ADPC): 5.2%), rise in ALT (ADPC: 4.3%), decrease in Cr (ADPC: − 2.5%), decrease in CRP (ADPC: − 15.4%), and decrease in LDH (− 2.5%) are signs of recovery. In contrast, a rise in LDH (3.9%) and a decreased ALT (− 12.1%) may predict a poor prognosis. However, the sample size of the two latter laboratory values was small, and the underlying comorbidity may explain, to some extent of, this poor prognosis. The full longitudinal course of laboratory values is illustrated in Fig. 3.

Consistent with this study, other studies found the trend of CRP^9,10,18^, LDH^10,11,13,18^, PLT^11,13^, lymphocyte^8,13^, urea^11,13^, and Cr can discriminate COVID-19 outcome. Our results fairly confirm results of Burke et al. study^13^ using dynamic time warping analysis. In contrast, WBC^11,13^, neutrophil count^8,11^, and AST^11^ were found to be beneficial for stratifying the risk of patients’ mortality, while we didn’t find this benefit. Zhao et al. used^11^ historical regression trees on 358 COVID-19 inpatients recruited in January 2020. The difference in sample size and trend analysis method may explain this contrast. Also, other biomarkers such as D-dimer^9,10^, ferritin^10^, interleukin-6^9,10^, troponin^10^, Prothrombin time (PT)^11^, and neutrophil-to-lymphocyte ratio^10,13^ had been proposed for prognosis prediction. Many of our biomarkers suggest that COVID-19 is a multi-organ disease, and their trend can reflect the improvement or deuteriation of COVID-19 patients.

The predictive value of laboratory examination on admission has been well established in the literature^6,7,19^. We used Lasso penalized regression method to increase the interpretation of proposed biomarkers and propose more accurate prognostic factors. We found a set of hematologic (WBC, neutrophil, lymphocyte, MCV, PCO2), inflammatory (CRP, D-dimer, LDH), renal function (Cr, BUN, K), liver function (alkaline phosphatase, bilirubin), coagulation (PT), and cardiac (CPK, CK-MB, Troponin) factors as indicators of poor prognosis. In our previous effort, we aimed to predict mortality risk by analyzing clinicolaboratory data collected upon admission. After selecting the most important prognostic factors and removing variables with collinearity, we identified ten laboratory exams as predictive factors, suggesting the role of laboratory exam as an independent predictor of mortality^20^. These findings show a multi-organ damage nature of COVID-19 infection.

Similar to our results, various studies showed that leukocytosis, neutrophilia^21,22^, and lymphopenia^23,24^ were mortality predictors. We found a higher prevalence of PLT abnormalities in non-survivors, comparable to a meta-analysis of 12 studies^25^. However, our LASSO regression analysis found that PLT is not an independent risk factor for mortality. Still, there are controversies, and further studies are needed. The neutrophils-to-lymphocyte ratio (a biomarker of systemic inflammatory response) was introduced as an independent biomarker of poor prognosis in COVID-19^26^. In contrast, in our study, the neutrophils-to-lymphocyte ratio was not confirmed as an independent risk factor, whereas it was significantly higher in non-survivors.

Several studies showed a significant correlation between COVID-19 infection, multiple organ involvement, and^27^ end-organ damage, leading to mortality. Therefore, several biomarkers related to organs’ function and end-organ damage have been investigated. Our results showed significant correlations between impairments in laboratory tests related to kidney (Cr, urea) and heart (CPK) with disease mortality. Consistent with our results, previous studies showed kidney impairment indicators on admission are associated with in-hospital mortality^28,29^. Similar to a study by Taj S. et al.^21^, our results indicate that elevations of transaminases are more common than increased bilirubin levels. However, interestingly we just found that direct and total bilirubin significantly correlates with COVID-19 patients’ mortality in our study.

Various studies have introduced abnormalities in coagulation parameters as prognostic factors, including elevated D-Dimer^30,31^ and prolonged PT^23^. Compatible with these studies, our results also confirm that in addition to coagulation dysfunction in COVID-19 patients, prolonged PT and increased D-dimer were predictors of mortality in these patients.

Studies showed electrolyte impairments in COVID-19 and suggest monitoring patients with electrolytes^32^. A study by Liu S et al. on 136 confirmed COVID-19 patients showed significantly increased 30-day mortality in COVID-19 patients with K levels ≥ 5.0 mmol/L^33^. Our results strongly support that electrolyte impairments are significantly higher in severe COVID-19 patients. Our analysis did not consider sodium (Na), calcium (Ca), magnesium (Mg), and phosphorus (P) impairments as mortality predictors. However, in line with the Liu S et al. study, our results show that a higher K level is strongly associated with COVID-19 mortality. We suggest accurate monitoring of K in COVID-19 patients to maintain it within normal ranges.

A pooled analysis of nine studies revealed that elevated LDH at the time of admission is associated with a 16-fold increase in odds of mortality in COVID-19 patients^34^. Our study supports significant differences between those who survived and non-survivors, and we introduce increased LDH as an independent risk factor for COVID-19 mortality. Despite these results, increased LDH did not influence our study’s 60-day mortality in COVID-19 patients, and we do not recommend this parameter be monitored in patients routinely.

Severe COVID-19 infection leads to the aggravation of inflammation, and studies recommend assessing cytokines to investigate excessive immune response. All inflammatory parameters studied in our survey, including cytokines, ESR, CRP, procalcitonin, and ferritin, were significantly elevated in non-survivors on admissions as expected, according to previous studies^21,22,35–37^. However, we only introduce CRP level as a potent predictor for mortality and suggest CRP monitoring to assess disease severity since it has a significant difference between those who survived and non-survivors in Cox regression and Kaplan–Meier survival analysis. In line with this result, various studies introduced elevated CRP levels at admission as a risk factor for mortality in COVID-19 patients^21,22,37,38^.

Many limitations need to be considered during the interpretation of this study. First, our hospitals were tertiary centers in Tehran and reached a maximum capacity during the peaks of COVID-19. Thus, more severe patients were admitted and have been investigated. Comorbidities were unavailable while it could enhance the study, and we could not minimize the confounding effect of the underlying diseases on laboratory parameters. Out-of-hospital mortality is not rare, and we could not account for that in our study. Since by the end of our study period, only about 1.08% of the total Iranian population had been fully vaccinated, only a few vaccinated patients were referred to the hospital. Therefore vaccinated patients were not included in the study^39^. Furthermore, the nature of retrospective studies minimizes the accuracy of predictive studies. In addition, patients with severe conditions have more laboratory examination, and this study’s high mortality rate also points to the selection of severe cases. Another issue is that the number of vaccinated individuals were rare in our study, and we didn’t capture their laboratory profile. The alpha and beta variants were known variants during study period, and the Delta and Omicron subtype of COVID-19 were not evident during the study period. Therefore, generalization of our result to vaccinated patients and other COVID-19 variants needs further validations in future studies.

In conclusion, this study proposed serial and admittance laboratory biomarkers to evaluate COVID-19 outcome. A novel approach had been taken to find prognostic markers in serial laboratory examinations. Careful attention and monitoring of COVID-19 patients’ laboratory results can help manage patients. Based on this large-scale study, serial examination of PLT, Cr, CRP, LDH, and ALT can guide healthcare professionals in monitoring patients. Moreover, disturbance in admittance levels of CBC, CRP, PCO2, Cr, BUN, potassium, PT, alkaline phosphatase, bilirubin, CPK, CK-MB, LDH, Troponin, and D-dimer increase the risk of mortality. Nevertheless, future studies are warranted to confirm the results of this retrospective study.

## Data Availability

The datasets used in the current study are available from the corresponding author on reasonable request. The dataset would be unreservedly available for use as a validation dataset of other research projects, after sending the request to the corresponding author (MAP), or SAASN. The data for Joinpoint analysis, code for data mining, and “Tehran COVID-19 Cohort” project information are available at https://github.com/Sdamirsa/COVID19_SerialLabratory and https://github.com/Sdamirsa/Tehran_COVID_Cohort.
